# Understanding Health Worker and Community Antibiotic Prescription-Adherence Practices for Acute Febrile Illness: A Nested Qualitative Study in the Shai-Osudoku District of Ghana and the Development of a Training-and-Communication Intervention

**DOI:** 10.1093/cid/ciad327

**Published:** 2023-07-25

**Authors:** Vida Ami Kukula, Selase Odopey, Emmanuel Arthur, Gabriel Odonkor, Elizabeth Awini, Alexander Adjei, Olawale Salami, Juvenal Nkeramahame, Philip Horgan, Piero Olliaro, John Williams, Rita Baiden

**Affiliations:** Social Science Department, Dodowa Health Research Centre, Dodowa, Ghana; Social Science Department, Dodowa Health Research Centre, Dodowa, Ghana; Social Science Department, Dodowa Health Research Centre, Dodowa, Ghana; Social Science Department, Dodowa Health Research Centre, Dodowa, Ghana; Social Science Department, Dodowa Health Research Centre, Dodowa, Ghana; Social Science Department, Dodowa Health Research Centre, Dodowa, Ghana; Department of Medicine, FIND, Geneva, Switzerland; Department of Medicine, FIND, Geneva, Switzerland; Department of Medicine, FIND, Geneva, Switzerland; Nuffield Department of Medicine, Big Data Institute, University of Oxford, Oxford, United Kingdom; Department of Medical Affairs, Evidence and Impact Oxford, Oxford, United Kingdom; Department of Medicine, FIND, Geneva, Switzerland; International Severe Acute Respiratory and Emerging Infection Consortium, Pandemic Sciences Institute, Nuffield Department of Medicine, University of Oxford, Oxford, United Kingdom; Social Science Department, Dodowa Health Research Centre, Dodowa, Ghana; Social Science Department, Dodowa Health Research Centre, Dodowa, Ghana

**Keywords:** acute febrile illness, antibiotics resistance, prescription adherence communication, prescription-adherence behavior

## Abstract

**Background:**

The aim was to explore behavioral factors relating to the prescription and communication of prescription-adherence messages for patients with acute febrile illness, from which to develop a training-and-communication (T&C) intervention to be delivered as part of a clinical trial.

**Methods:**

The study undertook a content analysis of primary, qualitative data collection using in-depth interviews and focus group discussions, informed by the Capability, Opportunity, Motivation (COM-B) theory of behavior, the Theoretical Domains Framework (TDF), and Behavior Change Wheel (BCW) approach, in health facilities (39 health workers) and communities (66 community members) in the Shai-Osudoku District of Ghana.

**Results:**

Health workers perceive that prescribers' and dispensers' communication with patients is influenced by the following factors: patient’s educational level, existing disease conditions, health worker's workload, patient's religion, language barrier between health worker and patient, outcome of laboratory results, and medicine availability. Community members’ adherence to prescription was influenced by the availability of money and affordability of medicine (outside of provision by the national health insurance scheme), the severity of the condition, work schedule, and forgetfulness.

**Conclusions:**

Our study contributes to knowledge on nesting qualitative methods in a clinical trial and reveals factors that affect the antibiotic prescription communication process. Tailored messages for patient-specific needs can shape antibiotic prescription adherence behavior and ultimately contribute to decreasing the incidence of antibiotic resistance.

## Teaser

This was a nested qualitative study to understand factors that influence healthcare providers’ prescription of, and patients’ adherence to, antibiotics. Emerging behavioral factors guided the development of a communication kit used in the intervention arm of the clinical trial.

Recognized as a global threat and a public health problem, antibiotic resistance is driven by antibiotic use [1]. Worldwide antibiotic use has increased considerably, by approximately 65% between 2000 and 2015 [1]. Much of this increase has been caused by the rapid expansion of antibiotic use in low- and middle-income countries (LMICs) [2]. Antibiotic consumption rates in many LMICs, including Ghana, are catching up with higher-income countries due to a lack of supportive regulation, just-in-case prescription of antibiotics, and widespread mal-prescribing practices such as informal use and adherence communication problems [2–5]. An array of factors influences inappropriate antibiotic prescription and use. These factors can be physician-related (eg, age, cadre, background, specialty, training, experience), patient-related (eg, sex, age, insurance status, comorbidities, income, knowledge, and expectations), and environmental dynamics (eg, access, support system, quality of care, and difficulties in diagnosis) [5–7].

The term “fever” in tropical areas is casually used to describe a spectrum of clinical manifestations of febrile illnesses [8]. In the current study, acute febrile illness is defined as fever with no focus or respiratory tract infection (RTI) lasting for no more than 7 days. In sub-Saharan Africa, antibiotics are normally used for viral infections like colds, upper respiratory infections, and other conditions in which antibiotics neither are appropriate nor resolve the illness. Although bacterial infections may require antibiotics, there is concern about their overuse and inappropriate use [4, 9]. Most health facilities in sub-Saharan Africa, including Ghana, lack the diagnostic capacity to identify acute febrile illnesses—a challenge, particularly in children and adolescents [10]. This misdirected and erroneous practice contributes to the inappropriate prescription of antibiotics; hence, the evolving agenda towards implementing interventions to ameliorate excessive and inappropriate antibiotic prescription and consumption [11].

Health systems have made efforts to revise antibiotic-prescribing practices, especially in developing countries [12]. In Ghana, the national-level approach to minimize antibiotic resistance in primary care includes regulating its use through the Ghana National Policy on Antimicrobial Use and Resistance through the introduction of the Standard Treatment Guidelines and Essential Medicines List [2, 13, 14]. The Ghana National Health Insurance Scheme (NHIS), with at least 16 million Ghanaians insured, covers many antibiotics for febrile illness and an NHIS-accredited facility that cannot offer a particular antibiotic due to stockouts is obliged to refer the patient to the next accredited facility that can prescribe or dispense the required medicine.

## THE DIAGNOSTIC USE ACCELERATOR STUDY

FIND's AMR Diagnostic Use Accelerator Program was launched at the AMR Call to Action event in Ghana on 18 November 2018. As part of the first stage of this program, a clinical trial (doi: 10.1186/s13063-020-04897-9) was deployed in several public health facilities in 5 countries; Ghana, Burkina Faso, and Uganda in Africa, India, and Nepal in Asia.

The trial involved both a quantitative component (a randomized controlled trial) and a nested qualitative component. The trial's primary objective was to assess the impact of a package consisting of diagnostic tools, clinical algorithms, clinical processes, and training and communication on (1) clinical outcomes and (2) antibiotic prescriptions compared with routine practices for children and adolescents presenting at outpatient clinics. The secondary study objectives were to assess adherence to (1) the new diagnostic algorithm by healthcare workers and (2) prescriptions by patients/caregivers and (3) to evaluate the safety outcomes of these practices.

The baseline qualitative study described here investigated the social and contextual behavior determinants of adherence to prescription and the communication of messages among community members and healthcare workers in the Shai-Osudoku District of Ghana. Based on the emerging determinants, a training-and-communication (T&C) package (included under Supplementary Table 1) was developed, consisting of training for healthcare workers and communication messages supporting prescription adherence. Training of healthcare workers took place before enrollment into the clinical component of the trial, with communication messages provided for intervention-arm patients, as 1 component of the intervention package.

## METHODS

### Study Design

#### Study Site and Participants

The nested qualitative study was conducted between November 2019 and December 2019, using in-depth interviews (IDIs) and focus group discussions (FGDs) among health workers and community members in the Shai-Osudoku District of Ghana. The study was conducted by authors V. K., S. O., and E. A. Author V. K. is a nurse researcher with extensive experience in qualitative research methods. Author S. O. is an early-career qualitative researcher. Author E. Arthur is a qualitative research officer. The primary investigators do not work in the health facilities mentioned in this study. Shai-Osudoku District is located in the Greater Accra Region of Ghana, with Dodowa as its capital town. The Dodowa Health Research Centre operates a health, demographic, and surveillance system covering a land area of 1528.9 km^2^ and has 136 906 registered residents living in 25 221 households.

#### Qualitative Methods: Why Qualitative Methods?

Qualitative research has proven useful when applied preceding or with randomized controlled trials to unravel enablers, barriers, and influencers of behavior [15, 16]. Qualitative research methods were used within the current trial to explore behavioral determinants and complexities of social, cultural, monetary, and peer influences on antibiotics practices.

Qualitative research methods broadly apply the interpretivism paradigm, which purports “to explore and understand the social world through the participants and their perspectives; and explanations can only be offered at the level of meaning rather than cause” [17].

We chose qualitative methods because they provide rationales and reasons for the specific phenomenon, theory, or choice [18]. In the current study, qualitative techniques enabled healthcare providers and community members to share their views and lived experiences on antibiotic prescription and adherence practices. The findings from the nested qualitative methods have influenced the trial intervention design, allowing for a fuller engagement with care receivers [19].

### Development of Topic Guides

Topic guides for use in Ghana were adapted, for local context, from templates developed for the study covering the different study countries. The design of topic guides was influenced by the Theoretical Domains Framework (TDF) [20] and the Capability, Opportunity, and Motivation model of behavior (COM-B) as described in “The Behavior Change Wheel” by Michie et al [21]. Specifically, prompts were developed around the core questions based on relevant categories in the TDF and COM-B models. Adaptions were made to the multisite templates to reflect local language expression and explore contextual enablers of and barriers to antibiotics prescription adherence and communication, including expanding the probes and improving conversational flow in IDIs or FGDs.

### Study Instruments Data-Collection Methods

We used both IDIs and FGDs to answer our research questions regarding [22]. Focus group discussions were held in groups of 6–12 participants to understand the contextual and social fabric and how these opinions are formed in the natural social contexts [22]. We elicited communitywide and health worker–group perspectives on antibiotics practices.

### Sampling and Saturation

Purposive sampling was used to select healthcare workers and community members to participate in the research to saturation.

#### Selection of Health Workers for Focus Group Discussions and In-Depth Interviews

All health workers in the district who prescribe and dispense medicines were included as potential participants in the study. These health workers included primary prescribers (doctors, midwives, physician assistants), dispensers (pharmacists), and pharmacy assistants. We purposively selected and contacted health workers (who prescribe and/or dispense antibiotics) by phone to participate in the study based on availability and their roles in prescribing and dispensing medicines in the district. This was done with assistance from the district health promotion officer at Shai-Osudoku District Health Directorate. The research team then sought their interest, availability, time, and place for the interview. A total of 39 healthcare providers participated in 2 FGDs (8 participants in each group) and 23 IDIs. Participants for each FGD and IDI comprised health workers from public and private health facilities, including 3 hospitals and 3 health centers within the district. Health workers who participated in this study were aged between 27 and 65 years and had a minimum work experience of 2 and a maximum of 39 years. Focus group discussions with health workers were conducted at the District Health Administration in Dodowa and Osudoku Health Centre in Asutsuare, and all IDIs were held at the health facilities. Data-collection activities using daily interview notes were reviewed after each day's work to determine information saturation. As data collection progressed, saturation level was reached after 2 FGDs and 23 IDIs as no new information emerged from the health worker's interviews. At this point, recruitment and data collection were stopped.

#### Selection of Community Members for Focus Group Discussions

Purposive sampling was used to select community members who had visited a health facility within the last 2 weeks, had been prescribed antibiotics, and were from different households. A community focal person helped the team identify, invite, and recruit eligible community members to participate in the study. Sixty-six community members partook in 7 FGDs (8–11 participants). A total of 5 FGDs were held with adult caregivers: 2 FGDs were held with 17 adult male caregivers and 3 FGDs were held with 31 female adult caregivers aged 24–45 years and 15–60 years, respectively. For the adolescents, 9 females and 9 males participated in 2 separate FGDs. The adolescents’ ages ranged from 15 to 18 years. The venues for the FGDs included classrooms, church halls, and open spaces near a riverbank. The environments were supportive for confidential discussions because they were quiet, airy, and with minimal distractions. The study team assisted the participants in setting ground rules to guide the conversation.

Saturation was reviewed at the end of each day of data collection. As data collection progressed, saturation level was reached after 7 FGDs as there was no new information emerging from the community member FGDs. At this point, recruitment and data collection were stopped.

### How Confidentiality Was Achieved

The IDI participants were informed that information shared with the study team would be used for the sole purpose of research. For the FGD participants, the study team entreated the discussants to desist from discussion and share all other group members’ views to individuals who did not participate in the FGD. This was a ground rule set in agreement with the FGD participants prior to the start of the discussion. Also, participants were identified using numbers; no identifiable information will be included when the quotes are used in our field reports or published work.

### Informed Consent Process

Based on the Informed Consent Form (ICF), the study team explained the nature of the research to the participant in either Twi, Ga-Dangme, or English, depending on the participant's language of choice. Consent was given by participants aged 18 years and above. For participants under 18 years, assent was sought and consent was given by their parents/guardians. Participants were informed that their participation was voluntary. Participants and study team members who were obtaining consent each signed or thumb-printed and dated a statement of informed consent that meets the requirements of the Ghana Health Service Ethics Review Committee. Participants had ample time to ask questions, receive answers, and consent. Illiterate participants provided a thumbprint on the ICF and the ICF was signed and dated by an impartial witness. A copy of the ICF(s) was given to the participants.

### Data Collection and Management

Data collection was conducted between November 2019 and January 2021 before the initiation of the intervention. Handwritten notes and audio recordings were taken for all IDIs and FGDs. The IDIs lasted approximately 1 hour, while FGDs were held for 1.5 to 2 hours.

During the study team debriefing sessions at the end of each day, audio recordings were downloaded onto a laptop, transcribed into Dangme and Twi, and translated verbatim into English. The audio recordings were then assigned to team members for transcription, and independent transcribers validated the transcripts. The debriefing sessions also enabled the team to take note of new probes for the subsequent interviews.

### Data Analysis

Content analysis [23] was used to analyze the audio transcriptions and handwritten notes from FDGs and IDIs. The first codes were identified from the first batch of 10 transcripts, influenced by the prompt topics in the topic guides, and compiled into a coding manual.

The coding of transcripts was conducted by authors V. K., S. O., and E. Awini. Differences in coding were resolved through team discussions.

Codes were organized into categories and subcategories that summarized and brought meaning to the text. Themes were identified by analyzing patterns and connections within and between categories using a manual matrix created by the study team and reviewed by the qualitative investigator. Within the matrices, all data that pertained to a particular theme were assembled, capturing the similarities and differences in participant responses.

**Figure 1.**
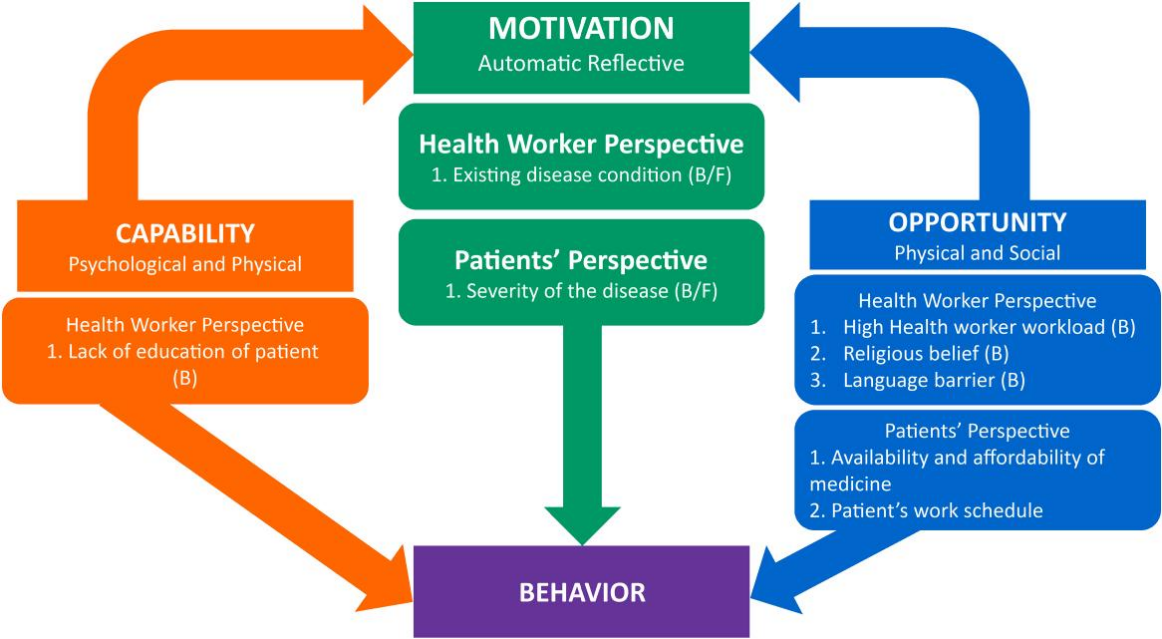
RCT behavior change model adapted from Michie et al [21] to explore behavioral factors relating to prescription and prescription-adherence messages for patients with acute febrile illness to develop a training-and-communication intervention to be delivered as part of a clinical trial. Abbreviations: B, barrier; F, facilitator; RCT, randomized controlled trial.

The data were structured into healthcare providers and community perspectives and further dividing them into behavioral “barriers” and “facilitators” where practicable. We mapped our analysis into the COM-B framework from the Behavior Change Wheel in order to establish applicable behavior change techniques for our intervention (Figure 1).

To optimize facilitators and overcome barriers towards achieving the objectives of the study, we used the COM-B framework. Capability barriers, for example, were addressed through education on prescription adherence; opportunity was tackled through addressing language barrier issues; and motivation barriers were tackled using communication and awareness on severity of disease. In addition to using the COM-B framework, we analyzed the data for contextual themes that may not be explicitly mapped onto the framework (eg, reliance on dispensers and associated cost of medicine) to help us understand issues of adherence.

The themes and connections were used to develop the T&C package for use within the clinical trial intervention package.

### Process to Develop the T&C Intervention Package From Research Findings

The T&C package was designed based on the behavior determinants identified from the baseline results. Each determinant was categorized as a barrier, enabler, or not influencing behavior. For each behavior determinant, specific messages were developed for prescribers to communicate adherence.

## RESULTS

### Health Worker Perspectives

The health workers stated that there is no standard written document for communicating antibiotic prescription adherence but advocated for its development. How and what prescribers and dispensers communicate is influenced by the patient’s educational level, existing disease condition, workload of health workers, patient's religion, language barrier between health worker and patient, the outcome of laboratory results, and medicine availability.

#### Patients’ Educational Level Affects Prescription Understanding and Forgetfulness

Prescribers and dispensers highlighted that if a patient/guardian is educated, grasping and understanding information on prescription adherence is easy. According to prescribers and dispensers, some patients even come to the health facility already having a lot of knowledge about antibiotics and the need to adhere; others ask a lot of questions, and this makes communication easier.

“I think it depends on how educated they [patients] are … because they will be asking you a lot of questions. ‘Doctor, why are you giving me this [medicine]?’ When you meet someone like that it actually makes you take your time to explain to them. So, I think because of their educational level, they know about some of these things, so they ask a lot of questions and some of them know about it. Okay, so when you meet such people, they tend to ask questions. So, the communication goes [well]. …”—IDI with a physician assistant

On the other hand, prescribers and dispensers agreed that for patients/guardians who are not educated, grasping information on antibiotic prescription adherence is very difficult and hence education during the consultation is time-consuming. Such patients sometimes forget all the instructions given; hence, adherence becomes difficult for them.

#### Blunt Messages on Existing Disease Condition

With regard to disease condition and laboratory results, some prescribers mentioned that their communication on prescription adherence is based on the severity of the disease condition, clinical findings, history presentation, physical examination, and sometimes laboratory investigation.

“We educate them based on the findings, thus the physical findings, including physical examination and the lab result, we let them know, maybe, there is an infection in the system so because of that we are giving you this antibiotic. If you adhere to the drug, you will be fine, if you don’t take it well, after 48 hours you may think you are fine, a week later you will come back, and we will have to give you the same drug again. And we also explain the side effects and the effects of not taking the drug accordingly. The body will build resistance and that is another major side effect, then it will get to a point the medication we give you will not work; you will have to buy a higher drug which is more expensive.”—IDI with a physician assistant

The health workers also sometimes must resort to threats when they realize the patient has a severe disease and does not adhere to instructions on the prescription, such as the following:

“If you do not take the medicine prescribed to you as instructed you may get reinfection, your disease may worsen, or you may die.”—IDI with a doctor

“I will tell you why you are supposed to be on the antibiotics for a woman who has maybe a pelvic inflammatory disease, I will tell you the antibiotics is for 14 days, you’re supposed to take it, if you don’t the effect of you having infertility, the scare alone makes them compliant.”—IDI with a doctor

#### Health Worker Workload

A prescriber's workload will result in not spending adequate time with their patients, shortening all forms of communication on prescription adherence, and omitting to communicate adherence.

“In this hospital, because we are getting an average 90 patients, you can’t spend 30 minutes on one patient so you just quickly skip, you go to how they should take it and sometimes even explaining to them the adverse effects, you don’t get time to do it so basically you just tell them when and where until they get that thing.”—IDI with a doctor

#### Influence of Religious Factors

Most often, patients hold back from accepting the prescription advice when prescribers and dispensers are educating them on prescription adherence during the consultation when this interferes with their preconceived ideas and beliefs. This makes it difficult for prescribers and dispensers to effectively give the right prescription-adherence information. Patients may resort to their pastors’ advice, prayers, and herbal medicines for their disease condition even though they have received information on prescription adherence. As a result, these patients will not adhere to instructions on prescriptions.

“I will say religious factors because we struggle a lot with ‘my pastor says this and then I am going for prayers here so it will go’. So, they think that after two days or the first day they should feel completely well and then when they don’t, they go to a prayer camp. The difficulty has to do with when they go and do herbs and then they come back worse off, and you have to start [treatment] all over again.”—IDI with a doctor

#### Language Barriers

What and how to communicate is also affected by language. When a prescriber is unable to speak the patient's local language, communication becomes a challenge. Sometimes the prescriber relies on the assistance of a colleague to communicate with the patient, which can be a breach of confidentiality.

“Sometimes it could be due to the language barrier. You know we are in a Dangme land. The area is dominated by Dangme-speaking people, and not all the staff understand their local dialect. Especially the aged, they don’t understand the other languages the staff also speak so you will have to get a third party who will have to explain, so while explaining you wouldn’t know whether what the third party is telling the client is exactly as you told the person or not. So, language is a factor.”—IDI with a doctor

#### Availability of Medicines at the Health Facility

##### Reliance on Dispensers

When medicines are not readily available at the health facility due to stockouts, prescribers and dispensers are not able to effectively communicate prescription adherence. This is because patients will have to purchase medicine at a store in their communities and some of these stores are not licensed and hence are not able to give appropriate instructions on adherence.

##### Associated Costs

Similarly, sometimes, patients end up not buying the medicines prescribed to them when not available at the health facility, because most medicines sold outside the health facilities are not covered by health insurance.

“Medicine availability can also be a factor because if they come and you don’t have the medicine, we tell them to buy them outside. So, we are not sure whether they will get the needed counseling on adherence at where they buy the medicine or not.”—IDI with a pharmacist

### Community Perspectives on Antibiotics Prescription and Adherence

Community members’ adherence to a prescription is influenced by the availability of money and the affordability of medicine, severity of the condition, ability to remember medicine instructions, ability to understand all instructions clearly, and strict supervision from family members and friends.

#### Availability and Affordability of Medicines

Study results showed that community members’ decision to purchase and adhere to antibiotic prescriptions is influenced by the availability and affordability of medicines at the health facility, without stockouts. Free or low-cost medicines available at the health facility make it easier for caregivers to have access to the prescribed medicines and thereby obtain information on prescription adherence as compared with buying outside the health facility.

“For me, if you come to the clinic, and they have shortage of drugs, it's important for them to write it for you to go and buy, but if the drug is available then they will give it out to you.”—FGD with female caregivers

Participants also mentioned that their ability to follow prescriptions depends on having enough money to afford the cost of medicines.

“I would have to give out money to the motor rider to and from, but if I don’t have money, I can’t buy the drug for my child.”—FGD with female caregiver

#### Fear of the Severity of the Condition

The severity of the disease condition is also one of the major factors motivating community members to purchase and adhere to antibiotic prescriptions from prescribers and dispensers. Caregivers perceive that the severity of the disease condition may lead to negative health outcomes such as death and paralysis. This situation causes fear and panic, thereby compelling caregivers to purchase medication and follow instructions on adherence.

“Maybe you think the disease is not severe since the child is playing but after you take him/her to the hospital and went to do lab, they saw that the illness can paralyze the child so when they prescribe a medicine, you have to give exactly how it was instructed so that the illness wouldn’t be severe. I remember taking my twins to the hospital, one was seriously sick, but that child was rather discharged that day and asked to be taken home but the other one who was not seriously sick was rather admitted.”—FGD with female caregivers

#### Patient's Work Schedule Interferes With Dosage Schedule

Community members complained that their work schedule also influences their decision to purchase and adhere to antibiotic prescriptions. Some said the nature of their work makes it difficult for them to complete the full course of medication and to follow instructions given to them by prescribers and dispensers. As a result, they tend not to adhere to antibiotic prescriptions.

“For instance, let's say I am a charcoal burner and I go to the bush to get woods, I go to the clinic and drugs are prescribed for me. Because I don’t have time, I will leave the medicines and will not take it. I won’t take it because maybe I will be going in the bush and so I will take one in the morning when going to the bush and when I come back in the evening, I would have been tired and forget about taking the medicine. So, I won’t take it and it will be there.”—FGD with adolescent

### Summary of Factors Affecting Adherence to Prescription and the Communication of Adherence Messages

We recorded consistent messages by community members and prescribers. They both pointed to drug availability at the health center and the associated extra costs and lack of communication by dispensers when medicines are bought outside of the health center. Community members highlight that costs and finances are important factors in buying the prescribed medicine.

Both patients and health workers highlighted that fear of the severity of illness motivates better prescription adherence. Additionally, health workers highlighted that the limited amount of time for consultations hindered good communication of prescription-adherence messages, education and language barriers were constraints, and preconceived religious beliefs or influence hindered the acceptance of adherence messages.

### Training-and-Communication Package

A T&C package was developed based on the themes/behavior determinants identified in the research findings. The T&C package consists of training for healthcare workers who will communicate messages of adherence to patients in the intervention arm of the clinical trial and the communication messages that are provided to the patients/caregivers. For each behavior determinant, the corresponding message to be given by the prescribers and dispensers during the trial phase of the study was provided. Patients in the intervention arm of the clinical trial are given these messages when antibiotics are prescribed to them. The training materials and communication messages can be found in Supplementary Table 1.

## DISCUSSION

We aimed to understand and describe social and contextual behavior determinants of adherence to prescription, and the communication of messages among community members and healthcare workers in the Shai-Osudoku District of Ghana. We then developed a communication tool that has a tailored message for each behavior adherence determinant identified that was used in the intervention arm of the clinical trial, alongside training for health workers in the delivery of the messages. Our study revealed that prescribers/dispensers communicate to patients depending on the patient's educational level, existing disease condition, workload, religion, medicine availability, and language. Community members’ adherence to a prescription is determined by the availability of money, affordability of medicine, severity of the condition, and work schedule (which translates to forgetfulness).

The outcome of laboratory results and the severity of the disease condition influence a provider's communication about prescription adherence. Explaining the importance of adhering to antibiotics to a patient with a severe condition or who has a negative laboratory outcome will make a provider caution a patient about the consequences of nonadherence. Patients are likely to take the medicine if they know the effects on their health outcome of nonadherence [7]. Our study showed that prescription-adherence communication is affected by a patient's educational level. An educated patient is more likely to ask questions and confidently seek clarification during consultation. Ineffective provider–patient communication can result in poor adherence to prescription instructions [7]. In addition to seeking help from a colleague fluent in the patient's language and considering the obstacles created by multiple dialects and illiteracy in our study population, we advocate using pictures to overcome barriers in communicating about adherence. In explaining contextual and behavioral determinants of health, religion is identified as influencing health-seeking behavior and medication adherence. In Ghana, where the majority of the population are deemed Christians, it emerged that patients will adhere to their pastor's instructions instead of following their physician's advice.

Our study also revealed that cost is a barrier to prescription adherence, related to either the availability of money or the affordability of the prescribed antibiotic. Antibiotics are provided under the NHIS, which the majority of Ghanaians are subscribed to. However, in unfortunate cases in which the medicines are not available at the health facility, our study shows that the patients have to purchase the medicine at a pharmacy or drug outlet in the community However, the NHIS requires the provider to refer the patients to another NHIS-accredited facility in such circumstances. It is, therefore, unfortunate that some NHIS members have to pay for medicines that are not covered by the scheme or buy them elsewhere. Sometimes the cost of acquiring the medicine increases because of the cost of transportation to find a pharmacy or licensed chemical sellers to buy from. Some people receive or buy the prescribed antibiotics but forget to ingest them at the provider's specified times because of their work schedules. Forgetting to take medicines will invariably result in misuse and possible resistance.

Workload is a potent but uncontrollable factor in how and what a prescriber communicates. Long queues and noisy hall walls/waiting areas can easily cause a prescriber/dispenser to rush through consultations. Opportunities to enforce or educate the patient on adherence, thereby straining the provider–patient communication, that is necessary to mitigate antibiotic resistance in our health system.

### Strengths and Limitations

Our study documents adherence practices in the Shai-Osudoku District of Ghana. This has allowed us to understand and develop a communication tool that best suits our study setting for the intervention arm of the clinical trial. Further integrating a qualitative study into a clinical trial has uncovered unique variations, behavior determinants, and the interconnection and influence of these on prescription adherence [24]. This has been particularly relevant in customizing the messages that patients in the intervention arm of the clinical trial receive. Although extensive communication of adherence to patients is abridged due to patient workload, prescribers and dispensers in this study benefit from guidance in communication to their patients using the T&C package in support of better health outcomes.

There are a few limitations to our study. Due to its qualitative nature, the findings we present are not generalizable and only relate to the setting we studied. Our study also missed possibly relevant information on prescription practices from informal dispensers, such as drug peddlers, because the T&C package was intended to serve the intervention arm of the clinical trial. It was impossible to address some behavior determinants through the training and communication package. For instance, when medicines are unavailable in the facility and patients had to buy them outside, even though prescribers will communicate prescription adherence, they are uncertain whether patients will purchase (and adhere to) the prescribed antibiotics. Despite the above issues, our study reveals key education areas that require enforcement when communicating on prescription adherence for antibiotics.

### Conclusions

Understanding what influences how a health worker will communicate antibiotics prescription adherence and how a patient/caregiver adheres to those instructions enabled the development of an intervention that best suits the intervention arm of our clinical trial. We agree that further studies that draw on perspectives of informal prescribers/dispensers, such as drug peddlers, will augment the appreciation of contextual issues that underpin consumption and, subsequently, adherence to antibiotics prescription in Ghana. This study is a useful guide for future multi-methods clinical trials in Ghana. Our health system will also benefit from continuous education, a novel context-driven prescription-adherence communication tool, and awareness of the dire effects of antibiotic resistance from nonadherence to prescription advice.

## Supplementary Data

Supplementary materials are available at *Clinical Infectious Diseases* online. Consisting of data provided by the authors to benefit the reader, the posted materials are not copyedited and are the sole responsibility of the authors, so questions or comments should be addressed to the corresponding author.

## Supplementary Material

ciad327_Supplementary_Data

## Notes

***Author contributions.*** Conceptualization: V. K., E. Arthur, and S. O. Analysis: S. O. and E. Arthur Writing: V. K. and S. O. Investigation: S. O. and E. Arthur Reviewing and editing: all authors.

***Disclaimer.*** The study funders had no role in data collection, interpretation, or reporting.

***Financial support*.** This work is supported by a contract between the Swiss Agency for Development and Cooperation, Global Health Division, and Foundation for Innovative New Diagnostics (FIND) (contract number 810571721 titled “Advancing access to better diagnostics for febrile childhood illnesses”); the Federal Ministry of Economic Cooperation and Development (BMZ); and a Memorandum of Understanding between the UK Government Secretary of State for Health and Social Care and FIND. FIND is also using core funding and internal resources to manage the program.

***Supplement sponsorship*.** This article appears as part of the supplement “Using Diagnostic Tools to Support Antimicrobial Stewardship and Improve Outcome in Resource-Limited Contexts,” sponsored by FIND.

***Ethical approval*.** The work described in this paper has received ethical approval as part of the approval of the overall trial protocol by the Oxford University Clinical Trials Research Ethics Committee (OxTREC number 52–19) and by the Ghana Health Service Ethics Review Committee (GHS-ERC 014/07/19).

## References

[1] Klein EY, Van Boeckel TP, Martinez EM, et al Global increase and geographic convergence in antibiotic consumption between 2000 and 2015. Proc Natl Acad Sci USA 2018; 115:E3463-70. 29581252 10.1073/pnas.1717295115 PMC5899442

[2] Opoku MM, Affran Bonful H, Koram KA. Antibiotic prescription for febrile outpatients: a health facility-based secondary data analysis for the greater Accra region of Ghana. BMC Health Serv Res 2020; 20:978. 33109158 10.1186/s12913-020-05771-9 PMC7590657

[3] Asante KP, Boamah EA, Abdulai MA, et al Knowledge of antibiotic resistance and antibiotic prescription practices among prescribers in the Brong Ahafo region of Ghana: a cross-sectional study. BMC Health Serv Res 2017; 17:422. 28633631 10.1186/s12913-017-2365-2 PMC5477684

[4] Batwala V, Magnussen P, Nuwaha F. Antibiotic use among patients with febrile illness in a low malaria endemicity setting in Uganda. Malar J 2011; 10:377. 22183039 10.1186/1475-2875-10-377 PMC3258227

[5] Strumann C, Steinhaeuser J, Emcke T, Sönnichsen A, Goetz K. Communication training and the prescribing pattern of antibiotic prescription in primary health care. PLoS One 2020; 15:e0233345. 32428012 10.1371/journal.pone.0233345 PMC7237035

[6] Sirota M, Round T, Samaranayaka S, Kostopoulou O. Expectations for antibiotics increase their prescribing: causal evidence about localized impact. Heal Psychol 2017; 36:402–9. 10.1037/hea0000456 28206788

[7] Jimmy B, Jose J. Patient medication adherence: measures in daily practice. Oman Med J 2011; 26:155–9. 22043406 10.5001/omj.2011.38 PMC3191684

[8] Bhaskaran D, Chadha SS, Sarin S, Sen R, Arafah S, Dittrich S. Diagnostic tools used in the evaluation of acute febrile illness in south India: a scoping review. BMC Infect Dis 2019; 19:970. 31722678 10.1186/s12879-019-4589-8 PMC6854686

[9] Apenteng JA, Addy BS, Onwukwe EO, Brookman-Amissah MG. Antibiotics prescribing patterns and incidence of respiratory tract infection in children under five years: a study in two hospitals in Accra, Ghana. Int J Med Med Sci 2018; 10:47–58.

[10] Kaboré B, Post A, Lompo P, et al Aetiology of acute febrile illness in children in a high malaria transmission area in West Africa. Clin Microbiol Infect 2021; 27:590–6. 32505586 10.1016/j.cmi.2020.05.029

[11] O’Connor R, O’Doherty J, O’Regan A, Dunne C. Antibiotic use for acute respiratory tract infections (ARTI) in primary care; what factors affect prescribing and why is it important? A narrative review. Ir J Med Sci 2018; 187:969–86. 29532292 10.1007/s11845-018-1774-5 PMC6209023

[12] Rahman N A, Teng CL, Sivasampu S. Antibiotic prescribing in public and private practice: a cross-sectional study in primary care clinics in Malaysia. BMC Infect Dis 2016; 16:208. 27188538 10.1186/s12879-016-1530-2 PMC4869350

[13] World Health Organization . WHO Global Strategy for Containment of Antimicrobial Resistance. Geneva, Switzerland: World Health Organization, 2001.

[14] Ministry of Health; Ministry of Food and Agriculture; Ministry of Environment Science Technology and Innovation; Ministry of Fisheries and Aquaculture Development. National Action Plan (NAP) for Antimicrobial Use and Resistance in Ghana. Ghana: Ghana National Action Plan for Antimicrobial Use and Resistance Ministry of Health Ministry of Food and Agriculture Ministry of Environment, Science, Technology and Innovation Ministry, 2017.

[15] Cooper C, O’Cathain A, Hind D, Adamson J, Lawton J, Baird W. Conducting qualitative research within clinical trials units: avoiding potential pitfalls. Contemp Clin Trials 2014; 38:338–43. 24937019 10.1016/j.cct.2014.06.002

[16] Clement C, Edwards SL, Rapport F, Russell IT, Hutchings HA. Exploring qualitative methods reported in registered trials and their yields (EQUITY): systematic review. Trials 2018; 19:589. 30373646 10.1186/s13063-018-2983-y PMC6206926

[17] Ritchie J, Lewis J, eds. Qualitative research practice: a guide for social science students and researchers. London: Sage Publications, 2003.

[18] Starks H, Trinidad SB. Choose your method: a comparison of phenomenology, discourse analysis, and grounded theory. Qual Health Res 2007; 17:1372–80. 18000076 10.1177/1049732307307031

[19] Rapport F, Clement C. Qualitative research within trials: developing a standard operating procedure for a clinical trials unit. Trials 2013; 14:54. 23433341 10.1186/1745-6215-14-54 PMC3599333

[20] Atkins L, Francis J, Islam R, et al A guide to using the theoretical domains framework of behaviour change to investigate implementation problems. Implement Sci 2017; 12:77. 28637486 10.1186/s13012-017-0605-9 PMC5480145

[21] Michie S, van Stralen MM, West R. The behaviour change wheel: a new method for characterising and designing behaviour change interventions. Implement Sci 2011; 6:42. 21513547 10.1186/1748-5908-6-42 PMC3096582

[22] Patton MQ, Cochran M. A guide to using qualitative research methodology. London: Patton Michael Quinn, 2002.

[23] Erlingsson CL, Brysiewicz P, Erlingsson C. A hands-on guide to doing content analysis. Artic African J Emerg Med 2017; 7:93–9. 10.1016/j.afjem.2017.08.001 PMC6234169 30456117

[24] Richards DA, Bazeley P, Borglin G, et al Integrating quantitative and qualitative data and findings when undertaking randomised controlled trials communication. BMJ Open 2019; 9:32081. 10.1136/bmjopen-2019-032081 PMC6886933 31772096

