# Supplementary material for: Understanding Health Worker and Community Antibiotic Prescription-Adherence Practices for Acute Febrile Illness: A Nested Qualitative Study in the Shai-Osudoku District of Ghana and the Development of a Training-and-Communication Intervention
Source: Clin Infect Dis. 2023 Jul 25;77(Suppl 2):S182–90. doi: 10.1093/cid/ciad327 (PMC10368414; doi:10.1093/cid/ciad327)
Supplement: ciad327_Supplementary_Data [file ciad327_supplementary_data.docx]

# Supplementary information

### Table 1. Linking Research Findings to Training and Communication Package

| Behavior determinant | Message to be delivered |
| --- | --- |
| High cost of prescription to patient/caregiver | Tell patient/caregivers their prescription is covered by the NHIS  It is very important for you to get this prescription. I suggest soliciting help from family members or friends. |
| High workload/little time of doctors | Doctors, pharmacist, pharmacy assistants and nurses should reinforce prescription and adherence message at each point of contact  I am reinforcing all that the doctor/nurse told you about your prescription today |
| Influence of Religion | It is what I saw in you and your laboratory results that has informed my prescription for you today. Please, adhere to the prescription I have given to you.  I understand you like prayers. It is important for you to take your medicines before or in parallel to prayers. |
| Forgetfulness | Make sure you have a family member who will always remind you to take your prescription every day and on time |
| Patients’ education level | Read the label on the medicine package/leaflet  Make sure you take all the antibiotics prescribed to you today |
| Severity of disease | Complete the full prescription even if you feel your condition has improved  Take your medicines just as prescribed paying attention to the time and number of tablets/spoonfuls  Do not share your antibiotic prescription with others  Do not use leftover medicines of others |
| Medicine Availability | You can get all the medicines prescribed you today at the facility’s pharmacy  Bring back the prescription of any medicine that is not available at the pharmacy |
| Language Barrier | Use infographics/visuals to support communicating the messages |
